# A smart thermo- and pH-responsive microfiltration membrane based on three-dimensional inverse colloidal crystals

**DOI:** 10.1038/s41598-017-12426-z

**Published:** 2017-09-21

**Authors:** Bing Yu, Qianqian Song, Hailin Cong, Xiaodan Xu, Dongwei Han, Zhongmin Geng, Xiaoyan Zhang, Muhammad Usman

**Affiliations:** 1 Institute of Biomedical Materials and Engineering, College of Chemistry and Chemical Engineering, Qingdao University, Qingdao, 266071 China; 2 Laboratory for New Fiber Materials and Modern Textile, Growing Base for State Key Laboratory, College of Materials Science and Engineering, Qingdao University, Qingdao, 266071 China

## Abstract

In this paper, a thermo- and pH-responsive microfiltration membrane was prepared based on three-dimensional (3D) inverse colloidal crystals (ICC). To manufacture the smart ICC membrane, the typical thermo-responsive N-isopropylacrylamide (NIPAM) and pH-responsive methacrylic acid (MAA) were polymerized inside silica colloidal crystals. The smart ICC membranes were characterized by SEM, IR and contact angle measurements. Moreover, the permeability of smart microfiltration membrane was carried out by the KCl diffusion tests. The result showed that effective diameter of the polymer ICC membrane can be reversible tuned by temperature and pH. Besides, the functional ICC membrane showed outstanding temperature- and pH-responsive gating property, which was applied to separate particles of different sizes. The savvy environment-responsive gating membranes have potential uses in filtration, separation, purification, sensor and other applications.

## Introduction

Natural gemstone opals with huge radiant auxiliary have significant iridescent structural colors which have regular crystalline arrays of highly monodisperse silica colloids. The marvellous optical execution of CCs emerges from Bragg diffraction of light in particular wavelength ranges due to the specific ordered structures. In this manner, colloidal spheres like silica and polystyrene with nano/micro-scale diameter can be self-assembled into artificial CCs with 3D ordered structure under specific conditions. It is easy to control the synthesis of artificial CC either by conforming molecule collaboration or under external fields compared with natural opals^1–7^. The CCs bring out blossom of various exceedingly situated nanostructure exhibits with applications of optics. One of the most representative 3D structures is photonic crystals (PCs). As the name recommends, the PCs are crystalline materials. The refractive index is periodically balanced on a length scale equivalent to the light wavelength of interest^8–13^. Another significant structure is three-dimensionally ordered macroporous (3DOM) structure, additionally called inverse opals is produced by the colloidal crystal templating strategy. The produced porosity in 3DOM is the result of inverted macroscopic ordered composites. Generally, a structure based on ordering uniform spheres (polystyrene, polymethyl methacrylate or silica) were synthesized at first. The desired precursor materials (metals, metal alloys, metal oxides, polymers, carbon or soft responsive hydrogels) were filled and set in the interstitial voids. Finally, the original CC templates were removed by calcination or extraction^14–19^. This handling method prompts the development of 3DOM structure with an open, between associated macropore structure and nano-sized wall components. The 3DOM structure with occasional pores ordered in three dimensions, substantial surface range and volume has been principally utilized as a part of photonic crystal devices, photocatalysts, microelectronics, chromatographic materials and sorbents^20–25^.

To date, numerous responsive PCs have been produced for detecting different external stimuli, like temperature, pH, humidity, solvent, light, ions and magnetic field^26–28^. Compared with responsive PCs, the filtration membranes based on 3DOM with tunable pore size were studied less and most promptly synthesized as thin film. By changing the silica CC template diameter, Wang *et al*. manufactured microfiltration membranes with different pore sizes and thickness^29^. Permeating fluxes have been resolved for layers with various pore sizes and hydrophilicities to further particle separation. According to atom transfer radical polymerization (ATRP), ICC ultrafiltration membranes were synthesized with tunable pore sizes by growing poly(polyethylene glycol-*co*- methacrylate) chains on the membrane surface based on above microfiltration membranes^30^. Surface modification may be used to convert a microporous inverse colloidal crystal membrane into an ultrafiltration membrane^27^.

**Figure 1:**
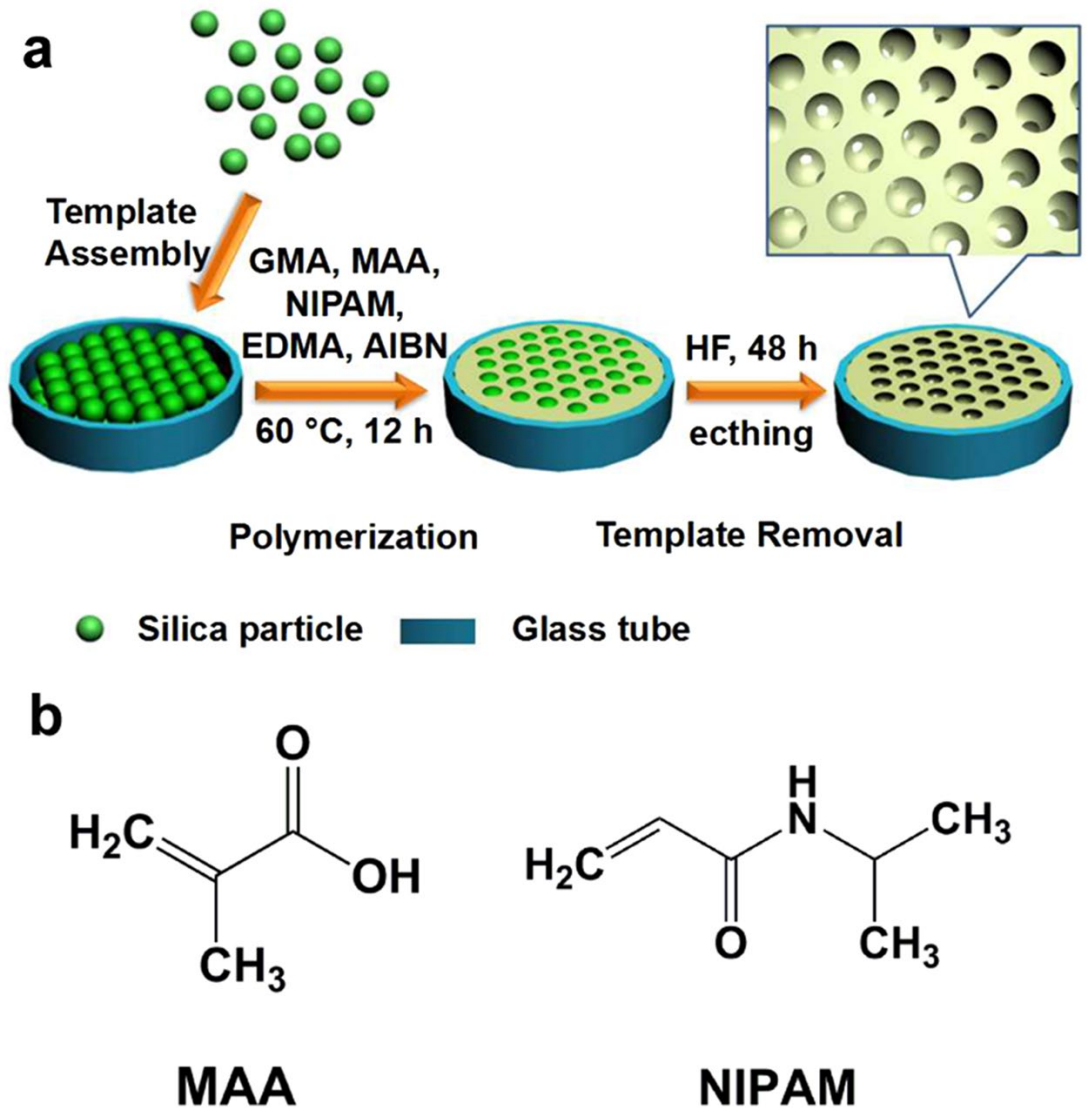
(**a**) Schematically illustration of the preparation of thermo-and pH-responsive ICC macroporous column; (**b**) Chemical structures of MAA and NIPAM.

Poly(*N*-isopropylacrylamide) (PNIPAM) is a typical thermal-responsive biocompatible polymer with a lower critical solution temperature (LCST) of 32 °C in water. Polymethacrylic acid (PMAA) is a typical pH-responsive biocompatible polymer with a pKa of 5.5. They have been generally utilized in the synthesis of smart gating membranes^31–33^. The chemical structures of the monomers are illustrated in Fig. 1. PNIPAM and PMAA chains are stretched when the temperature below LCST and pH > pKa due to the formation of hydrogen bonds with water, resulting in close state of the membrane. When the temperature is higher than LCST and pH < pKa, these chains are shrunken due to the broken of hydrogen bonds with water, resulting in open state of the membrane. In this paper, we resolved to build up a kind of thermo- and pH-responsive micro/nano filtration membrane based on ICC structure. It can filter nanoparticles with different diameters by changing temperature and pH. The fabrication and application of ICC filtration membranes as well as the ion and nanoparticles transportation properties of the obtained membrane were studied and discussed prelimarily. The colloidal crystal templating method typically requires three sequential steps^34–37^ as shown in Fig. 1a: (i) a CC template is prepared by ordering monodisperse silica microspheres into a face-centered close-packed structure in a glass tube, (ii) voids in the colloidal crystal are filled with thermo- and pH-responsive liquid polymer precursors which polymerized in interstices of the sphere templates, and (iii) an ICC column is produced after removing the template by HF etching. The structure which consists of a skeleton surrounding uniform close-packed macropores and nanopores was synthesized using this method.

## Results and Discussion

**Figure 2:**
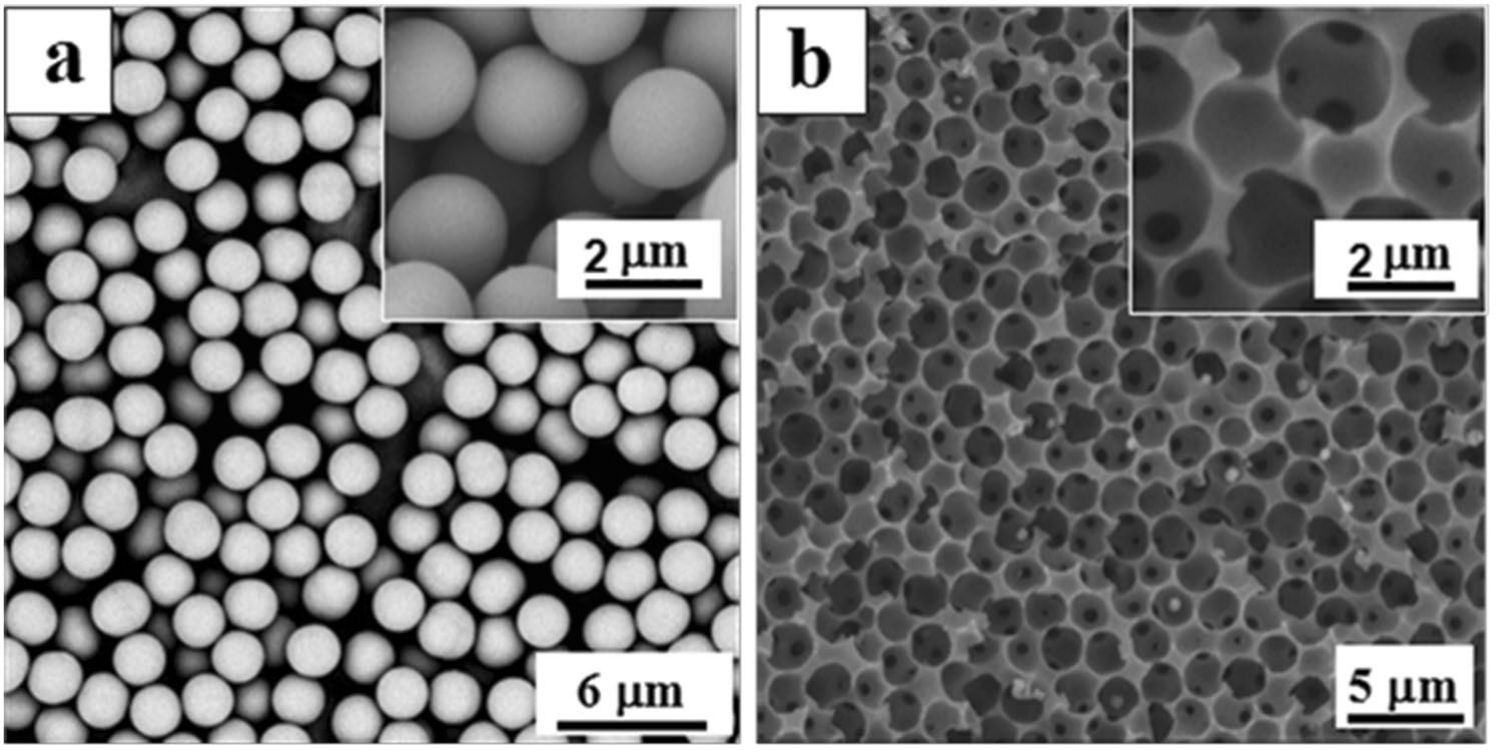
SEM image of SiO_2_ microspheres (**a**) and cross-sections of the ICC column (**b**), the inset images are amplified images of (**a**) and (**b**), respectively.

Figure 2 Shows SEM images of silica monodisperse microspheres with a diameter of 2 µm and P(NIPAM-MAA-GMA) inverse colloidal crystal (ICC) macroporous column. As shown in Fig. S1, it can be observed that the synthesized microspheres silica microspheres has good monodispersity and do not adhere to each other. It can be seen from Fig. 2b that ICC macroporous film has through-hole structure with ~2 µm big holes on the top layer and 440 ± 10 nm small holes underneath.

**Figure 3:**
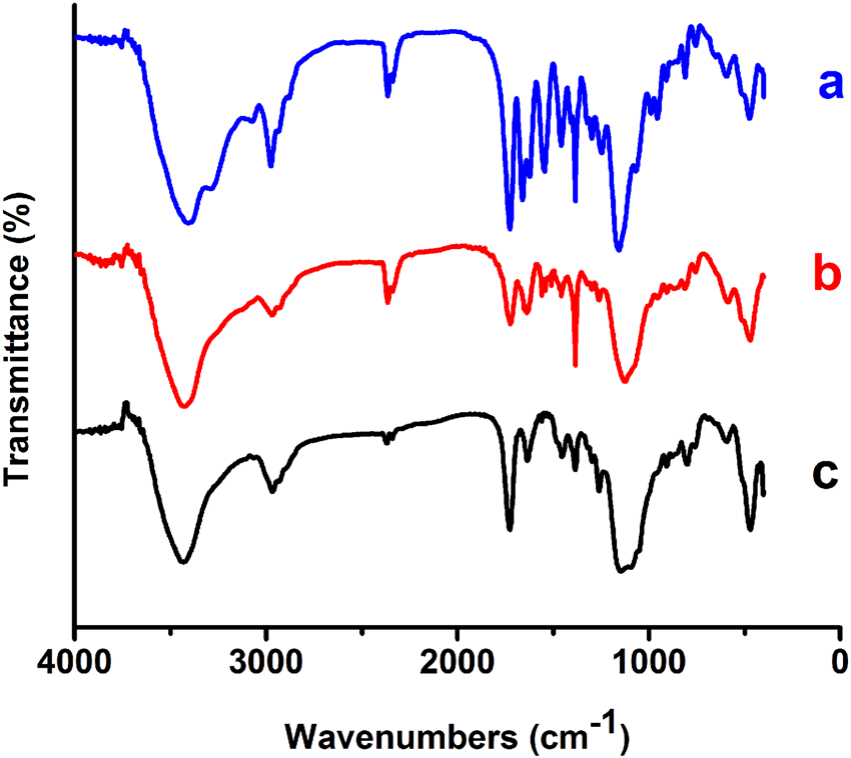
IR spectra of the co-polymers P(NIPAM-MAA-GMA) contains 50% (**a**), 30% (**b**) and 0% (**c**) of NIPAM and MAA.

Figure 3 shows the changes in IR spectra among the polymers with various contents of PNIPAM and PMAA. In all spectra a broad N-H stretching peak at 3423.1 cm^−1^ could be observed. Peaks at 2800~3100 cm^−1^ were assigned to the absorption peaks of C-H in CH_3_ and CH_2_. Two characteristic peaks of ester at 1161.0 cm^−1^ and 1080.0 cm^−1^ confirmed the GMA monomer. Compared to the IR spectrum of 0% NIPAM and MAA (black), 50% and 30% NIPAM and MAA (blue and red), the presence of secondary acyl group at 1646.9 cm^−1^ and 1544.7 cm^−1^ emerged from C=O stretching and N-H stretching of the amide group respectively which proved the presence of monomer NIPAM. Absorbance of 1385 cm^−1^ of -C(CH_3_)_2_ group further evidenced the existence of NIPAM. At the meanwhile, the presence of the hydroxyl and carboxyl groups at 3270 cm^−1^ and 1720 cm^−1^ arose from O-H and C=O stretching of the carboxylic acid, which confirmed the existence of monomer MAA. The results verified the successful copolymerization between monomers NIPAM, MAA and GMA. In addition, the different strength of peaks of the secondary acyl group at 1646.9 cm^−1^ and 1544.7 cm^−1^ confirmed different ratio of the NIPAM and MAA in P(NIPAM-MAA-GMA) co-polymers.

To investigate the surface wettability of the co-polymers ICC columns, the hydrophilicity of the columns with different content of NIPAM has been studied by contact angle measurements. As shown in Fig. S2, the hydrophilicity of the segments can be affected by the substance of NIPAM at the same temperature, because the PNIPAM is hydrophilic at low temperature and becomes hydrophobic at high temperature.

**Figure 4:**
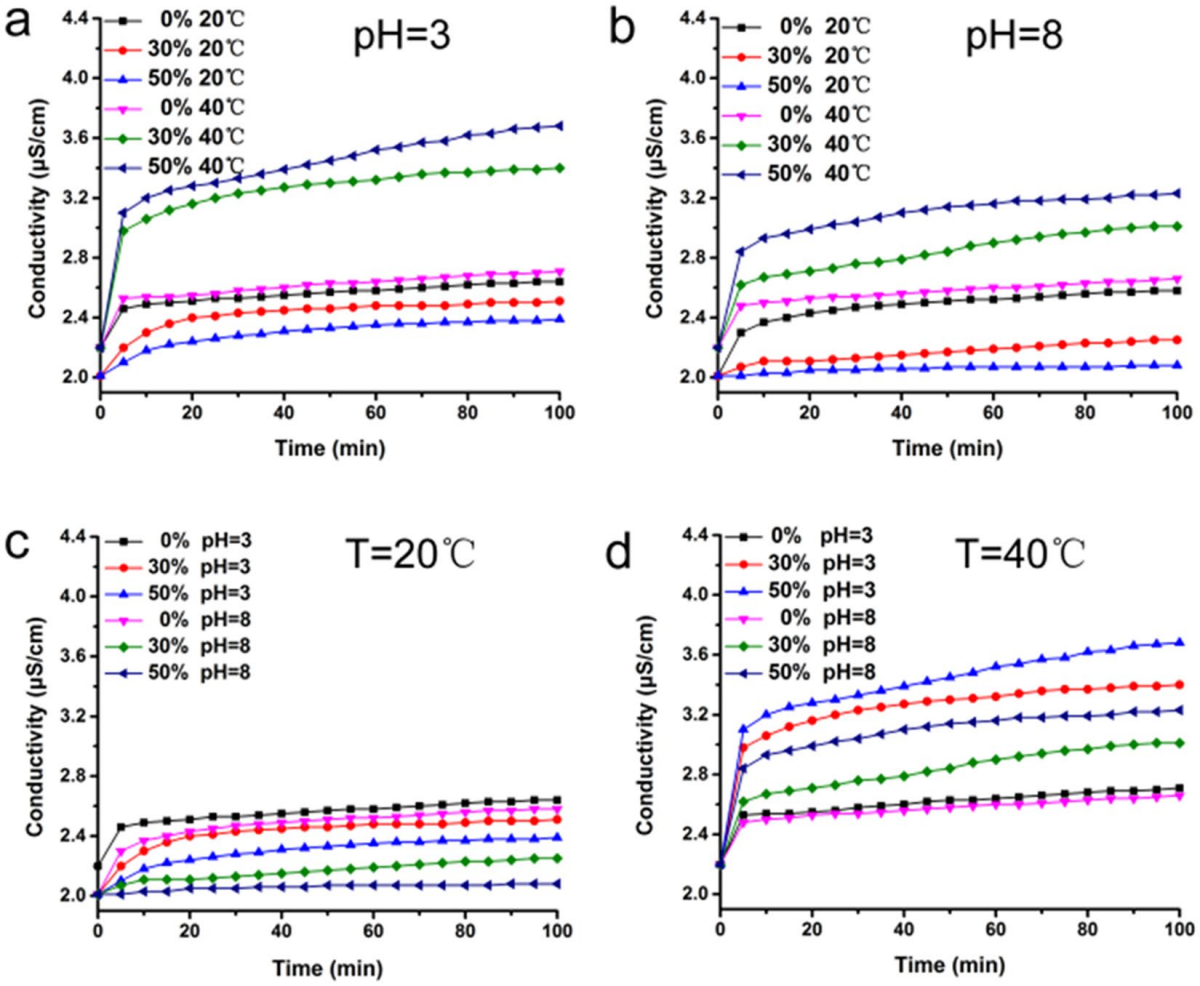
Ion transportation properties of the ICC membrane with different NIPAM and MAA content at different temperatures and pH values measured through conductivity variation in the downstream.

In order to evaluate the permeability of the ICC columns, we construct an instrument to measure ion transportation properties of the membrane (Fig. S3b). Briefly, the characterization of porous permeability could be assessed by measuring conductivity. In this experiment, two different concentrations of KCl solutions are connected only by the ICC macroporous column membrane. The conductivity change will be produced by the diffusion of K^+^ and Cl^−^ through the membrane driven by the concentration grads. As shown in Fig. 4a,b, the conductivity of P(NIPAM-MAA-GMA)−30 and P(NIPAM-MAA-GMA)−50 decrease at 20 °C, while an obviously variation can be observed at 40 °C. The variety of temperature-responsive conductivity performs in steady at various environments with various pH values. When the substance of NIPAM increases, the thermo-sensitive property with shrinking or stretching effect of NIPAM chains in ICC column get to be more stronger. This prompts a smaller or bigger pores at 20 °C or 40 °C. Therefore, P(NIPAM-MAA-GMA)−50 has smallest and largest conductivity than others at 20 °C and 40 °C. This phenomenon confirmed that we prepared the thermo-sensitive porous ICC membrane successfully. The conductivity of PGMA at different temperatures show no significant change. which further demonstrated the thermo-responsive ability of P(NIPAM-MAA-GMA). In the same way, the conductivity of P(NIPAM-MAA-GMA)−30 and P(NIPAM-MAA-GMA)−50 at pH = 8 is lower than that at pH = 3. At the point when the pH value changes from 3 to 8, the shrinking or stretching effect of MAA chains in ICC columns become stronger which lead to smaller or larger pores. The change trends of pH-responsive conductivity perform is consistent at different temperatures. However, conductivity of P(NIPAM-MAA-GMA)−30 and P(NIPAM-MAA-GMA)−50 at 40 °C is much higher than that at 20 °C which illustrated that thermo-sensitivity influence larger than pH-sensitivity comes from the most content of temperature-responsive NIPAM. This phenomenon confirmed that we synthesized the thermo- and pH-sensitive porous ICC membrane successfully. The conductivity of PGMA at environments with different pH values show no significant change which further demonstrated the pH-responsive ability of P(NIPAM-MAA-GMA).

**Figure 5:**
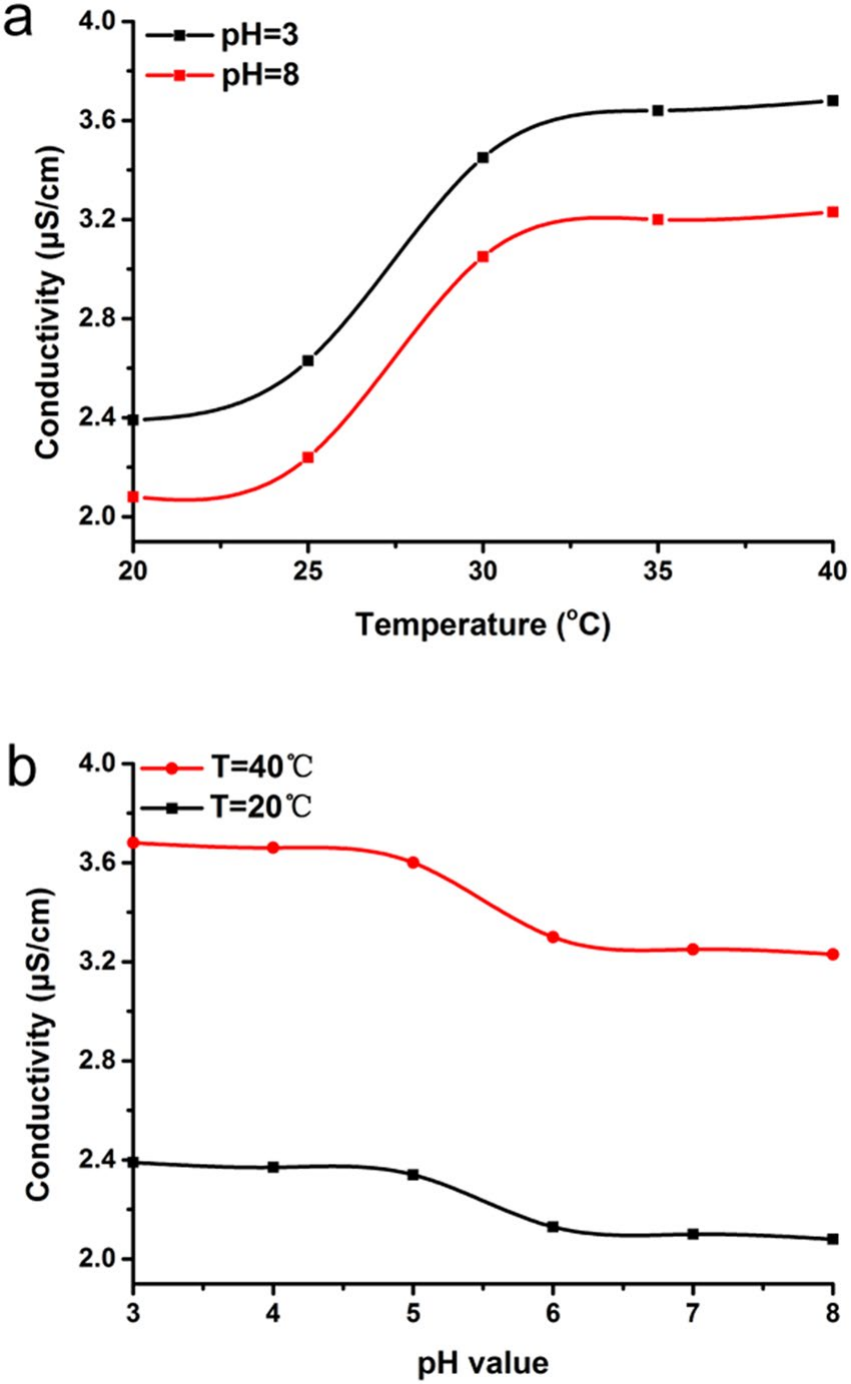
Ion transportation properties of the P(NIPAM-MAA-GMA)−50 ICC membrane at different temperatures and pH values measured through conductivity variation in the downstream.

As shown in Fig. 5a, with increasing of the temperature from 20 °C to 40 °C, the conductivity value of the P(NIPAM-MAA-GMA)−50 column had a rise abruptly at around 30 °C. This demonstrated the typical and great thermo-sensitive property. Due to thermo-responsive property of the PNIPAM polymers, when the temperature increases from 20 to 40 °C, the polymer shrank and led to the expansion of the channels and voids, resulting in higher conductivity. The reason about this move may presumably be that when the temperature below 30 °C, the polymer chains extended due to hydrogen bonding between the hydrophilic groups and water molecules. When the temperature exceeds 30 °C, thermal breakage of the hydrogen bonding led to shrinkage of the polymer chains and exclusion of water from the macroporous material, which led to the expanding behavior of the pores^28^. Likely, as shown in Fig. 5b, with increasing of the pH value from 3 to 8, the conductivity value of the P(NIPAM-MAA-GMA)−50 column decreases abruptly at pH = 5~6 which indicated that the typical and great pH-responsive property. At the point when the pH exceeds MAA’s pKa with about 5.5, the polymer chains expanded because hydrogen bonding between the hydrophilic groups and water molecules, which led to lower conductivity. When the pH value below pKa, pH-responsive breakage of the hydrogen bonding led to shrinkage of the polymer chains and exclusion of water from the macroporous material, which led to the expanding behavior of the pores and higher conductivity.

**Figure 6:**
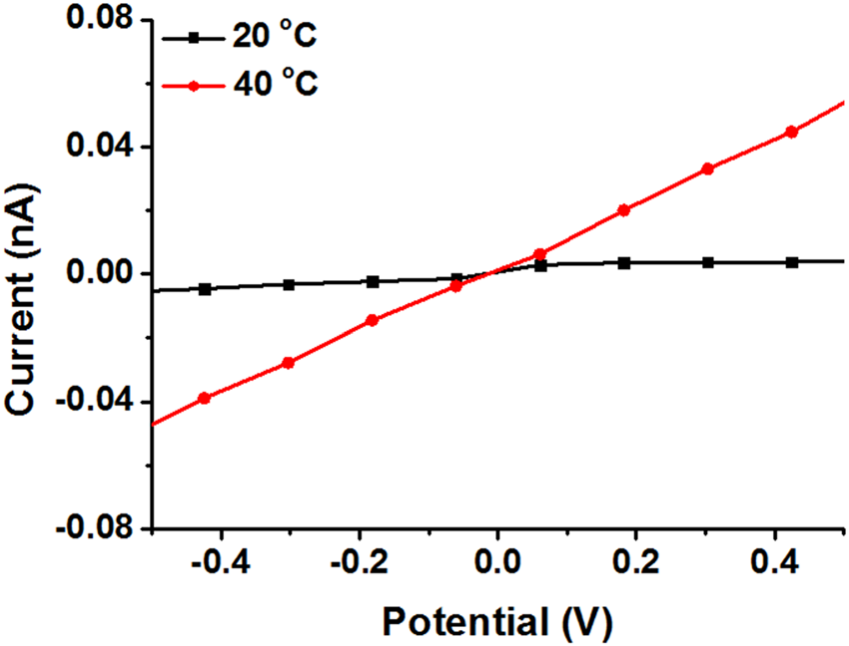
Current-voltage curve of P(NIPAM-MAA-GMA)−50 ICC column membrane changes with temperature.

The trans-film permeability can be correlated to the effective pore sizes by Hagen-Poiseuille’s law, as shown in equation (1)^38^:

1 $$Q=\frac{\pi {R}^{4}{\rm{\Delta }}P}{8\eta L}$$

where, *Q* stands for column flux, *R* represents the radius of the pore, ${\rm{\Delta }}P$ is trans-column pressure, *L* is the length of the pore channel, and *η* is the solution viscosity. Moreover, *R* could be estimated by equation (2):

2 $$R={(\frac{8Q\eta L}{\pi {\rm{\Delta }}P})}^{1/4}$$

based on the Hagen-Poiseuille’s equation: assuming macroporous column pore was cylindrical and the column length was not changed, the radius ratio of pores at 20 and 40 °C could be estimated via equation (3):

3 $$\frac{D}{{D}^{\text{'}}}=\frac{R}{{R}^{\text{'}}}={(\frac{\eta Q}{{\eta }^{\text{'}}{Q}^{\text{'}}})}^{1/4}\approx {(\frac{\eta K}{{\eta }^{\text{'}}{K}^{\text{'}}})}^{1/4}$$

where, *D* represents the diameter of the pore, *K* represents the conductivity. We know that the diameter of PGMA ICC column pore was 440 nm at 20 °C, Because of the low coefficient of thermal expansion of PGMA polymer, the pore diameter at 40 °C can also be considered as ~440 nm. Given the statics from Fig. 6c,d, pH doesn’t have much effect on the conductivity of PGMA ICC column membrane compared to temperature. Hence, we hypothesized that the diameter of PGMA ICC column pore is 440 nm under the above experimental conditions and focused on the effect of temperature. In order to estimate the contributions to the specific conductivity changes as temperature variations, we proceeded to estimate the column pore diameters considering the data in Fig. 6. For instance, as for the calculation of P(NIPAM-MAA-GMA)−50 ICC column pores’ diameter at 20 °C (*D′*) and pH = 3, we had known that *D* was 440 nm, *K* is the final conductivity values over time. Thus, the diameter of P(NIPAM-MAA-GMA)−50 ICC column pores can be calculated is 429 nm at 20 °C according to equation (3), and similarly, the diameter of P(NIPAM-MAA-GMA)−50 column can be obtained with 475 nm at 40 °C, which demonstrates the controllable modified ICC column pore sizes via temperature sensitive PNIPAM. The other computational data are summarized in Table 1. These results clearly evidence the nanovalving behavior of the temperature sensitive PNIPAM and pH sensitive PMAA in the copolymer membranes.

**Table 1:** Computational pore diameters of different membranes.

P(NIPAM-MAA) Content(%)	Diameters at 20 °C (nm)		Diameters at 40 °C (nm)	
	pH = 3	pH = 8	pH = 3	pH = 8
0	440	440	440	440
30	434	425	466	454
50	429	417	475	462

Figure 6 Shows the current-voltage curve of P(NIPAM-MAA-GMA)−50 ICC column membrane obtained at different temperatures. Obviously, the membrane exhibited a linear I-V characteristic. At 20 °C, PNIPAM segments stayed swollen, subsquently diminishing the powerful cross area of the pores. This was described by the low slope of the I-V curve, which was connected with a low conductance of the pores. Raising the temperature above the LCST promotes drastic changed on the conformational state of the PNIPAM segments. In this case, the segments suffered a transition into collapsed state, which also had an increasing impact on the effective diameter of the pores.

**Figure 7:**
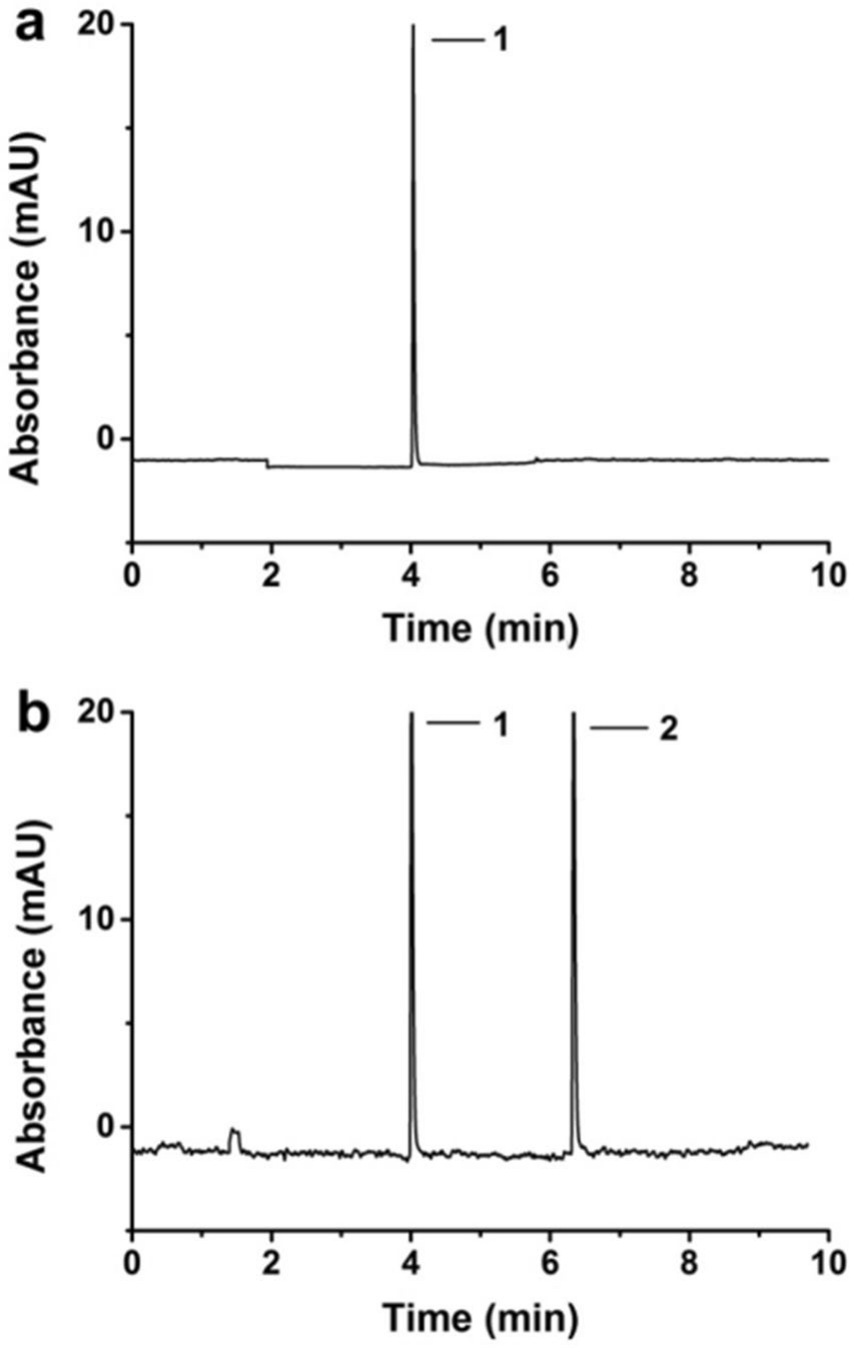
Capillary electrophoresis separation of filtrated polystyrene particles with different diameters at (**a**) T = 20 °C (**b**) T = 40 °C. Separation conditions: buffer, 40 mM phosphate (pH = 3.0); injection, 20 s with a height difference of 20 cm; applied voltage, +15 kV; UV detection, 254 nm; sample, 0.5 mg·mL^−1^ for each protein; capillary, 75 μm id × 50 cm (40 cm effective); capillary temperature, 25 °C. Peak identification: (1) 100 nm; (2) 450 nm.

Figure 7 shows capillary electrophoresis separation results for two polystyrene particles (100 and 450 nm) which were filtrated through the thermo- and pH-responsive P(NIPAM-MAA-GMA)−50 ICC column membrane at 20 °C (a) and 40 °C (b) in the optimized conditions. As discussed above, the P(NIPAM-MAA-GMA)−50 ICC column membrane indicated thermo-controllable diameters at various trial temperatures that the diameter is about 429 nm and 475 nm at 20 °C and 40 °C at pH = 3, respectively. Hence, the membrane based on ICC column could be used as a microfiltration membrane to isolate distinctive sizes of particles. The P(NIPAM-MAA-GMA)−50 ICC column membrane has smaller diameter at 20 °C which could be filtrate smaller polystyrene particles only (Fig. 7a) while the diameter of P(NIPAM-MAA-GMA)−50 ICC membrane at 40 °C became larger, and both polystyrene particles could be filtrated (Fig. 7b). Thus, this kind of smart thermo-sensitive film based on the ICC column can be utilized to isolate diverse sizes of small particles by tuning temperature, which has a potential usage in microfiltration applications.

**Figure 8:**
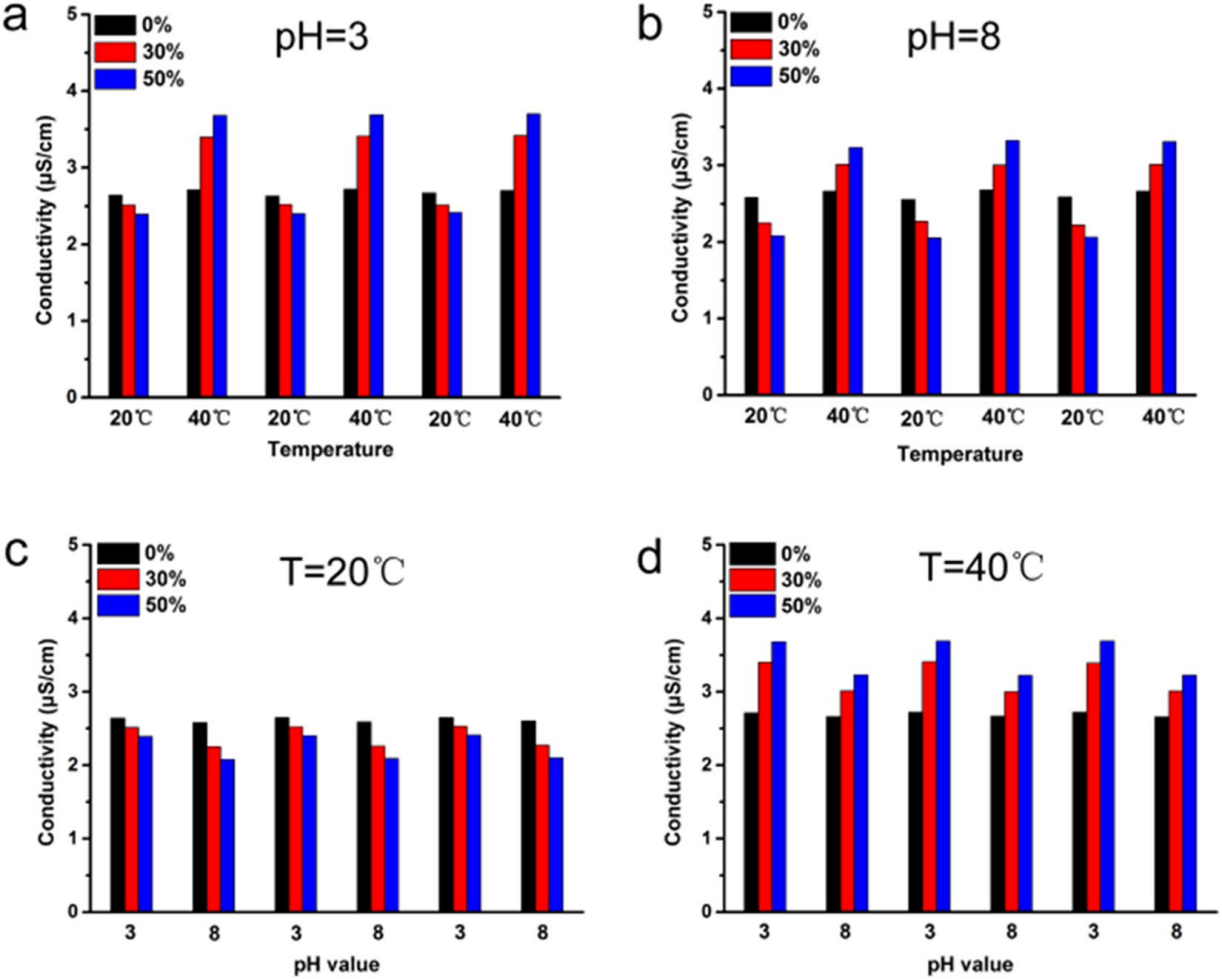
Stability and reproducibility of P(NIPAM-MAA-GMA) ICC column membrane.

In order to examine the reproducibility and stability of the thermal- and pH-responsive ICC membranes, the experiments for measuring conductivity were repeated at the same condition (Fig. 8). The conductivity value of the ICC column at typical time increased with increasing the temperature from 20 to 40 °C and decreased with decreasing the temperature from 40 to 20 °C at pH = 3 (Fig. 8a) and pH = 8 (Fig. 8b), which showed great reversible temperature-responsive property of the smart ICC column. At the same time, the conductivity value of the ICC column at typical time increased with changing the pH value from 8 to 3 and decreased with changing the pH value from 3 to 8 at 20 °C (Fig. 8c) and 40 °C (Fig. 8d), which showed excellent reversible pH-responsive property of ICC column. The outcomes demonstrated that the thermal- and pH-responsive ICC membranes have good reproducibility and stability.

## Methods

### Materials

Potassium chloride (KCl), hydrofluoric acid (HF) and 2,2′-azobis(2-methylpropion amidine) dihydrochloride (AIBN) were purchased from Tianjin Hengxing Chemical Reagent Company. Tetramethoxysilane (TEOS), ammonia solution (NH_3_·H_2_O), ethanol, N-isopropylacrylamide (NIPAM), methacrylic acid (MAA), glycidyl methacrylate (GMA) and ethylene glycol dimethacrylate (EDMA) were all obtained from Aladdin Industrial Corporation. Polydimethylsiloxane (PDMS) 184 and PDMS 184 curing agent were purchased from Dow Corning. All the chemicals were utilized as received unless noted elsewhere.

### Synthesis of monodisperse SiO2 microspheres

The SiO_2_ microspheres were prepared with a modified Stöber protocol^39^. 0.015 g of KCl was dissolved in the mixture of 60 mL double-distilled water and 54.0 g ethanol. Then, 3.0 mL ammonia solution was added in above liquid under stirring at 300 rpm at 30 °C (solution I). Under the condition of 0.2 mL/min velocity of flow, solution II which contained 35 mL ethanol and 4.0 g TEOS with an ultrasonically treated for 15 min was gradually injected into the above solution I. After 5 h, the diameter about 2.0 µm SiO_2_ microspheres could be synthesized and the obtained microspheres were purified by centrifugation and washed three times with ethanol. Then SiO_2_ microspheres were dried under vacuum at ambient temperature for 48 h.

### Preparation of silica colloidal crystal template

Silica colloidal crystal template was synthesized using a solvent centrifugal deposition process. The glass tube washed with alcohol and distilled water in an ultrasonic cleaner and dried at 70 °C for 6 h. SiO_2_ microspheres were uniformly dispersed in ethanol. Then it can be assembled in the glass tube by centrifugal deposition at 10 krpm for 30 min. Finally, the SiO_2_ microspheres were immobilized in the glass tube perfectly with typical face-centered cubic (fcc) packing through drying ethanol at 60 °C for 24 h.

### Preparation of ICC column membrane

Thermo- and pH-responsive ICC column with various levelled macroporous and nanoporous structures were fabricated through two steps. In the first step, silica colloidal crystal templates were simultaneously infiltrated with three monomers which were NIPAM, MAA and GMA, while EDMA acted as cross linking agent and AIBN acted as initiator. The weight ratio of PGMA column contained 0% NIPAM and 0% MAA was NIPAM: MAA: GMA: EDMA: AIBN = 0: 0: 0.3: 0.3: 0.01. Likely, the molar ratio of P(NIPAM-MAA-GMA)−30 column contained 30% NIPAM and MAA was NIPAM: MAA: GMA: EDMA: AIBN = 0.09: 0.01: 0.2: 0.3: 0.01, the molar ratio of P(NIPAM-MAA-GMA)−50 column contained 50% NIPAM and MAA was NIPAM: MAA: GMA: EDMA: AIBN = 0.14: 0.01: 0.15: 0.3: 0.01. The polymerization reaction proceeded for 12 h at 55 °C. Then, in the second step, in order to remove silica colloidal crystal template completely, the polymer column was taken out from glass tube and immerged in HF solution for 48 h. At last, the obtained ICC column was washed with ethanol and distilled water for three times to remove HF and other impurities.

### Instruments

The morphology and size of the acquired ICC structure and SiO_2_ particles were characterized by scanning electron microscopy (SEM, JEOL JSM-6309LV). The functional groups of PGMA, P(NIPAM-MAA-GMA)−30, P(NIPAM-MAA-GMA)−50 were determined by infrared spectroscopy (Spectrum 100, PerkinElmer). Hydrodynamic diameter of the SiO_2_ particles were measured by dynamic light scattering (DLS). The contact angle measurements were carried out by video contact angle meter (JY-82, Chengde Dingsheng). For the purpose of characterize the permeate property of the column, we manufactured a device to measure the changes of KCl conductivity. The liquid PDMS prepolymer and its curing agent (10: 1) mixing evenly were degassed for 15 min in vacuum and cast onto the glass wafer. Then it cured at 80 °C for 40 min. After the PDMS film cooling down, cutting out the film with appropriated size, and the column which prepared above was putted into the central hole of PDMS film tightly (Fig. S2a). The PDMS film was put in two plastic conductivity vessels through two elastic rubber sheets and clipped tightly. All the holes are aligned directly. Two KCl solutions with different conductivity were put into two conductivity vessels respectively. So the permeability of the porous can be obtained by measuring the changes in conductivity of the KCl solutions. The one plastic conductivity vessel was filled with high concentration KCl solutions (10^−4^ mol/L) and another one was filled with low concentration (10^−6^ mol/L). Meanwhile, the liquid level was keeping horizontal so that both ends of the channel’s hydraulic pressure of inconsistent conditions could be excluded. After the PDMS film, two rubber sheets and two plastic conductivity tanks bound tightly (Fig. S2b), we observed that the conductivity of lower concentration through the conductivity meter (DOS-11A, Shengci Instrument, Shanghai). Current-voltage measurements were taken subsequently to investigate the electrochemical properties of the column. It was mounted between halves of a conductivity cell, and each half-cell was filled with 1 mol/L KCl solution. Ag/AgCl electrodes were settled in each half-cell to apply desired trans-membrane potential and to measure the resulting ionic current. The current was measured with a picoammeter/voltage source (2634B, Keithley Instruments), a scanning voltage from −0.4 to 0.4 V on the one of membrane side was applied.

## Conclusions

In summary, the highly ordered permeable polymer columns were successfully synthesized by using SiO_2_ colloidal crystals as the templates. By introducing the thermo-responsive PNIPAM and pH-responsive PMAA into the membrane, the structures exhibited smart temperature- and pH-responsive property. The permeability of the column in response to changes in temperature and pH value was investigated by measuring the conductivity of KCl solutions. At the point when the temperature increased from 20 to 40 °C or the pH decreased from 8 to 3, the polymer shrank and prompted the development of the microchannels, bringing about higher conductivity. The conductivity decreased due to drop in temperature or rise in pH owning to the stretching of PNIPAM and PMAA chains and segments. The synthetic strategy which developed in this work could be utilized as a part of microfiltration, separation, purification, sensor and other applications.

## Electronic supplementary material

Supplementary information
