# Supplementary material for: A smart thermo- and pH-responsive microfiltration membrane based on three-dimensional inverse colloidal crystals
Source: Sci Rep. 2017 Sep 21;7:12112. doi: 10.1038/s41598-017-12426-z (PMC5608716; doi:10.1038/s41598-017-12426-z)
Supplement: Supplementary file 1 — Supplementary information [file 41598_2017_12426_MOESM1_ESM.doc]

**Supplementary Information**

**A smart thermo- and pH-responsive microfiltration membrane based on three-dimensional inverse colloidal crystals**

Bing Yu^1,2^, Qianqian Song^1^, Hailin Cong^1,2*^, Xiaodan Xu^1^, Dongwei Han^1^, Zhongmin Geng^1^, Xiaoyan Zhang^1^ , Muhammad Usman^1^

^1^Institute of Biomedical Materials and Engineering, College of Chemistry and Chemical Engineering, Qingdao University, Qingdao 266071, China

^2^Laboratory for New Fiber Materials and Modern Textile, Growing Base for State Key Laboratory, College of Materials Science and Engineering, Qingdao University, Qingdao 266071, China

^*^hailincong@yahoo.com

As shown in Figure. S1, we can see that the diameter of silica monodisperse is 2 µm, It can be observed that the synthesized microspheres silica microspheres has good monodispersity.





**Figure S1.** Hydrodynamic diameters of silica monodisperse determined by DLS.

As shown in Figure. S2, the contact angle of the water droplets on PGMA, P(NIPAM-MAA-GMA)-30, P(NIPAM-MAA-GMA)-50 is 51.03°, 45.73°, and 41.73° at 20 ºC, respectively. The values decrease as the content of NIPAM increases which means the hydrophilicity increases as the NIPAM’s content increases. At 40 °C, water droplets on PGMA, P(NIPAM-MAA-GMA)-30, P(NIPAM-MAA-GMA)-50 have contact angles of 41.59°, 44.92° and 45.28°, respectively. The hydrophilicity of the polymer decreases as the NIPAM’s content increases. From the results, we can acquire that the hydrophilicity of the segments can be affected by the substance of NIPAM at the same temperature, because the PNIPAM is hydrophilic at low temperature and becomes hydrophobic at high temperature.


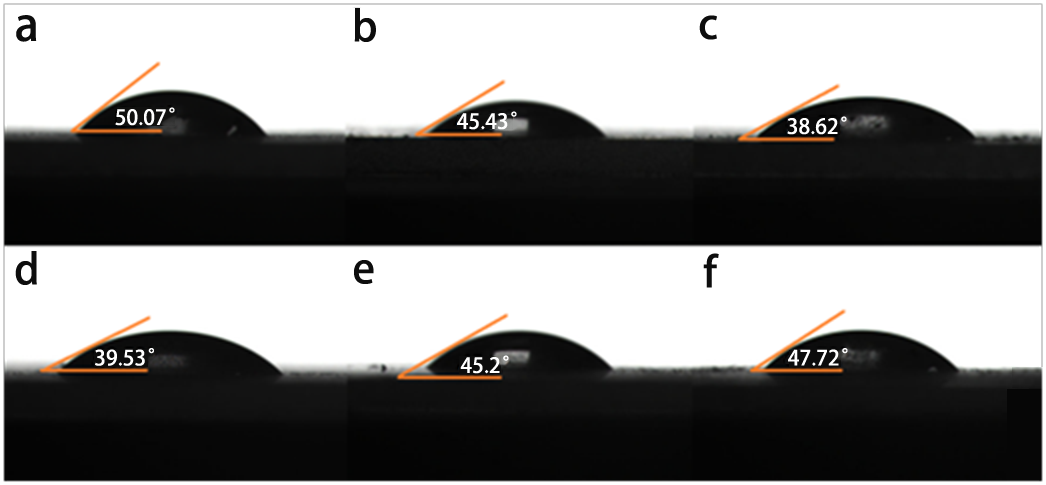


**Figure S2.** Contact angle measurements of the co-polymers P(NIPAM-MAA-GMA), contact angle of (a) 0, (b) 30%, (c) 50% NIPAM contained copolymers at 20 ˚C, (d) 0, (e) 30%, (f) 50% NIPAM contained copolymers at 40 ˚C.

The photographs of PDMS membrane with inverse macroporous column in the middle and the equipment for measuring ion transportation properties of the membrane were appeared in Figure. S3a and S3b, respectively. The ICC column diameter is ~4 mm and thick is ~2mm.


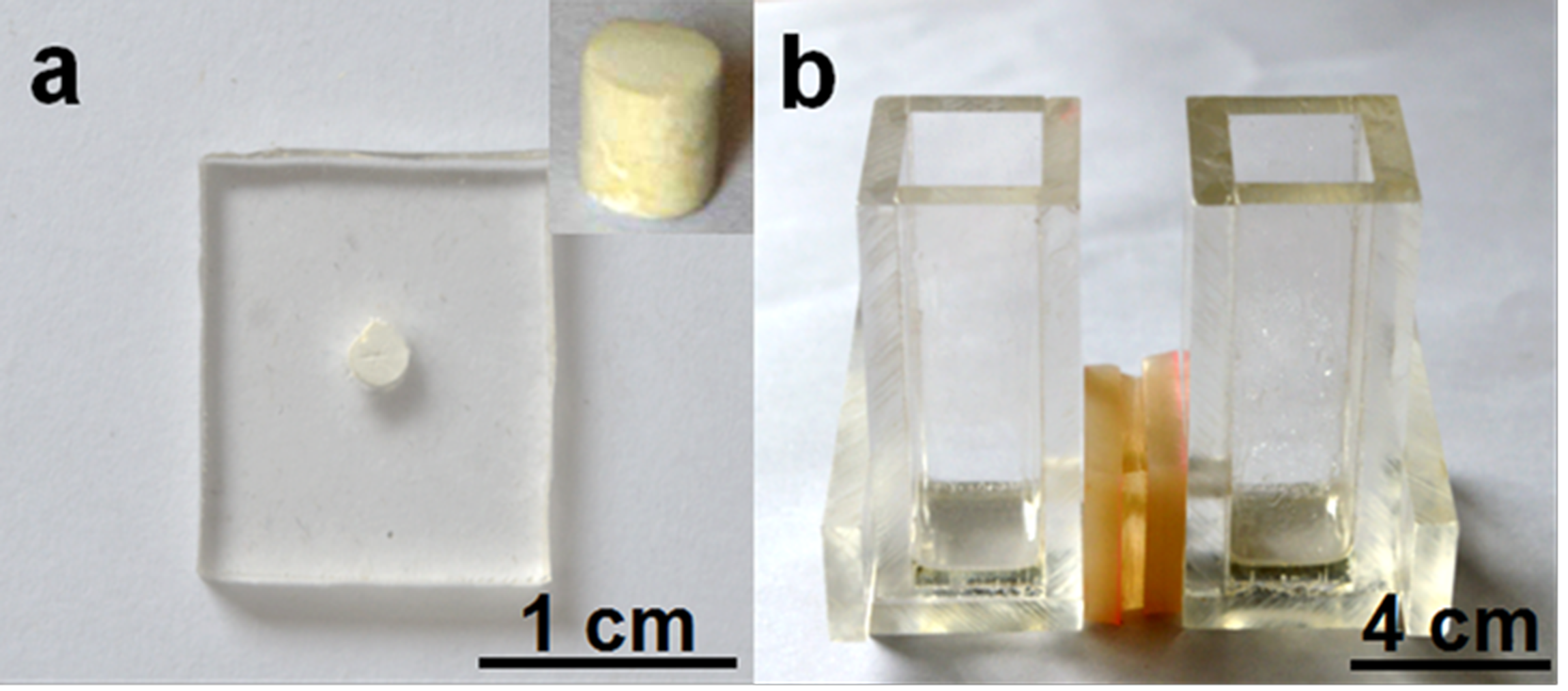


**Figure S3.** The pictures of PDMS membrane with inverse macroporous column (inset image) in the middle (a) and the equipment for measuring ion transportation properties of the membrane (b).
